# Mesalamine for Colorectal Cancer Prevention Programme in Lynch syndrome (MesaCAPP): a multicentre, multinational, randomised, two-arm, double-blind, phase II clinical study with mesalamine or placebo in carriers with Lynch syndrome – a study protocol

**DOI:** 10.1136/bmjopen-2025-100082

**Published:** 2025-11-09

**Authors:** Ann-Sofie Backman, Alexander Frank, Lars Joachim Lindberg, David Ljungman, Gustav Silander, Rita J Gustafsson, Tünde Bozsó, Peter T Schmidt, Michael Ingre, Martina Mittlbock, Christian Löwbeer, Jan Marsal, Annika Lindblom, Emma Tham, Christina Therkildsen, Christoph Gasche, The International MesaCAPP Study Group, Johannes Blom

**Affiliations:** 1 Dept. of Medicine, Karolinska Institutet, Stockholm, Sweden; 2 Dept. of Medicine, Ersta Hospital, Stockholm, Sweden; 3 Department of Medicine, Huddinge, Karolinska Institute, Stockholm, Sweden; 4 Department of Clinical Medicine, University of Copenhagen, Copenhagen, Denmark; 5 Copenhagen University Hospital, Copenhagen, Denmark; 6 Dept. of Surgery, University of Gothenburg, Gothenburg, Sweden; 7 Sahlgrenska Academy, Gothenburg, Västra Götaland County, Sweden; 8 Dept. of Radiation Sciences, Umeå University, Umeå, Sweden; 9 Norrlands University hospital, Uppsala, Austria; 10 Lund University, Lund, Sweden; 11 Skåne University Hospital, Malmö, Sweden; 12 Dept. of Medical Sciences, Uppsala University, Uppsala, Sweden; 13 Uppsala University Hospital, Uppsala, Sweden; 14 Medizinische Universitat Wien, Wien, Austria; 15 Dept. of Clinical Science and Education, Södersjukhuset, Stockholm, Sweden; 16 Molecular Medicine and Surgery, Karolinska Institute, Stockholm, Sweden; 17 Dept. of Molecular Medicine and Surgery, Karolinska Institutet, Stockholm, Sweden; 18 Dept. of Clinical Genetics and Genomics, Karolinska University Hospital, Stockholm, Sweden; 19 Clinical Research Department, Copenhagen University Hospital, Kobenhavn, Denmark; 20 Department of Surgical Gastroenterology, Copenhagen University Hospital, Kobenhavn, Denmark; 21 Med Univ Vienna, Vienna, UK

**Keywords:** Clinical Protocols, Clinical trials, Gastrointestinal tumours, Endoscopy, Microbiota

## Abstract

**Introduction:**

Lynch syndrome (LS) carriers have a 20–46% lifetime risk of colorectal cancer (CRC) due to mismatch repair gene variants. Mesalamine (5-ASA, 5-aminosalicylic acid), used safely in patients with ulcerative colitis, may reduce CRC risk in LS by decreasing microsatellite instability, a key driver of LS-related cancer. This study evaluates 5-ASA’s efficacy as a tolerable chemopreventive drug, aiming to improve long-term CRC prevention in LS.

**Methods and analysis:**

This multicentre, multinational, randomised, double-blind, two-arm, phase II clinical study will compare the effects of a 2-year daily intake of 5-ASA (2000 mg) to placebo in LS carriers. The primary objective is to assess whether mesalamine reduces colorectal neoplasia, both benign and malignant, compared with placebo in LS carriers, as detected by colonoscopy at the end of the treatment period (24 months±1 month) and on study completion. Secondary objectives include evaluating whether 5-ASA reduces neoplasia/tumour multiplicity and progression compared with placebo at specified time points, examining variations in the effects of 5-ASA versus placebo based on cancer history, sex and age (<45 years vs ≥45 years), and assessing the safety of 5-ASA in LS carriers.

**Ethics and dissemination:**

The trial is currently open for enrolment, having received ethical approval from the Regional Ethical Review Board in Stockholm and funding from the Swedish Research Council. The study protocol is the finalised V.10.0 (11 April 2024), transitioned to the European Clinical Trials Information System. LS remains underdiagnosed, which may limit recruitment. The results are of global interest and will be published in peer-reviewed journals and presented at scientific conferences.

**Trial registration number:**

ClinicalTrials.gov: NCT04920149. EudraCT: 2019-003011-55. EU CT: 2024-514765-19-01.

**STRENGTHS AND LIMITATIONS OF THIS STUDY**

- A multicentre, randomised, double-blind, placebo-controlled, phase II trial with prospective surveillance delivers a rigorous framework and provides a unique opportunity to evaluate chemopreventive efficacy in LS.
- Genotype-defined LS carriers (*MLH1*, *MSH2/EPCAM* or *MSH6;* ACMG class 4/5), with genotype prespecified in analyses, that is, covariate/interaction, rather than as a randomisation stratum.
- Structured long-term follow-up with serial biospecimen collection enables high-resolution, exploratory, pharmacodynamic, microbiome and inflammatory biomarker analyses under harmonised standard operating procedures.
- Recruiting this rare, genotype-defined cohort across Nordic centres strengthens generalisability, with coordinated oversight—positioning the trial to deliver reliable, broadly applicable evidence.
- With n=150 and observed event rates, precision for modest effects and subgroup estimates is limited; however, the study retains robust power for the end of treatment primary analysis.

## Introduction

 Colorectal cancer (CRC) is the third leading cause of cancer-related deaths in both males and females.^1^ Lynch syndrome (LS) is characterised by deleterious variants in DNA mismatch repair (MMR) genes—*MLH1*, *MSH2*, *MSH6*, *PMS2* or *EPCAM*—carries a 20–46% lifetime risk of CRC, despite regular colonoscopy surveillance. The penetrance and variable expressivity of LS-associated gene variants exhibit variability.^2^ The prevalence of LS among patients with colorectal and endometrial cancer is approximately 1–4%. When extrapolated to the general population, the estimated prevalence of LS ranges from 1 in 2000 to 1 in 660, with higher estimates suggesting a prevalence as frequent as 1 in 279 individuals.^3^ This implies that up to 1.6 million individuals may be affected within the European Union.

Cancer prevention in LS has been previously studied, as chemoprevention represents a major hope for LS family members. The CaPP2 trial tested the effect of 600 mg of aspirin (acetylsalicylic acid, ASA) in a large multinational cohort, showing no benefit for its primary outcome.^4^ However, a secondary post-hoc analysis demonstrated a statistically significant delayed effect.^5^ Additionally, a study published in 2020 consolidated the findings of the prior study, further validating the earlier results.^6^ A limitation of this study cohort was the inclusion of patients without a verified variant in an MMR gene, potentially complicating data analysis and the interpretation of subsequent conclusions. Recognising the challenges of long-term tolerance to 600 mg of ASA and the potential efficacy of lower doses with comparable chemopreventive effects, the CaPP2 trial authors have initiated a follow-up study, CaPP3, to compare lower doses of ASA with 600 mg. However, even lower doses are associated with an increased risk of gastrointestinal ulcers and bleeding, presenting challenges for promoting long-term adherence across all age groups.

MMR germline mutations, combined with somatic inactivation of the second allele, impair the cell’s ability to repair mismatch errors during DNA replication. This can lead to cancer through the accumulation of mutations that inactivate tumour suppressor genes or activate oncogenes. Additionally, defective MMR impairs the correction of errors in small repetitive DNA sequences, known as microsatellites, resulting in microsatellite instability (MSI), a hallmark of defective MMR.^7^ A potential strategy to impede cancer development in LS is to enhance replication fidelity, thereby reducing the occurrence of MSI.^8^ In vitro studies have demonstrated that mesalamine (5-ASA), a well-tolerated drug used for over 30 years in the treatment of ulcerative colitis, can reduce MSI by improving replication fidelity. 5-ASA activates a replication checkpoint, providing additional time for cells to progress through the S-phase, thereby reducing replication errors.^9^ The effect of 5-ASA is specific to the position of the amino group,^10^ as neither 3-ASA nor 4-ASA exhibited this effect. This mechanism applies to various repetitive sequences, tetranucleotide, dinucleotide and mononucleotide repeats, such as those found in *TGFBR2* and *ACVR2*.^11 12^ In Msh2^loxP/loxP^ Villin-Cre mice, which closely mimic LS in the intestine, 5-ASA reduced tumour incidence, tumour multiplicity and the number of variants in five selected microsatellite repeats in normal mucosa.^13^ Notably, this chemopreventive effect was not observed with aspirin which does not influence MSI.^8 14^

Regular surveillance can reduce the cancer burden by enabling early detection when the disease is still in a curable stage. The study population undergoes routine colonoscopy with polypectomy at frequent intervals, starting at an early age in accordance with national guidelines. Complications from colonoscopies, particularly during polypectomy of small polyps in the right-sided colon, along with procedural discomfort, may affect patient compliance. Despite frequent surveillance, LS carriers have been shown to develop CRCs, although these are typically detected at earlier stages.^1^ Any intervention that reduces this burden could provide significant health benefits. 5-ASA, at the proposed dose, has been well tolerated in clinical trials^15 16^ and in routine clinical practice for over 30 years. In ulcerative colitis, long-term treatment with 5-ASA has been associated with a reduced risk of CRC.^17^ Effective chemoprevention is likely to require lifelong medication in otherwise healthy individuals. To ensure compliance and maximise the benefit-risk balance, chemopreventive drugs should have minimal short- or long-term adverse effects. 5-ASA appears to be an ideal candidate for LS carriers. This study has the potential to establish a new cancer-preventive treatment option for LS carriers, contributing to more individualised care.

## Methods and analysis

### Primary objective

The primary objective is to test whether 5-ASA reduces the occurrence of any colorectal neoplasia (both benign and malignant tumours) compared with placebo in LS carriers, as detected by any colonoscopy until the end of treatment (EOT) (24 months+3 months) and end of study.

### Secondary objectives

- To evaluate whether 5-ASA reduces the number of colorectal neoplasms, including both benign and malignant tumours (tumour multiplicity), and prevents tumour progression compared with placebo in LS carriers at defined time points. Advanced adenomas are characterised by a diameter greater than 1 cm, villous or tubulovillous histology or high-grade dysplasia.
- To assess the safety profile of 5-ASA in LS carriers.
- To investigate metabolic, inflammatory and genomic biomarkers associated with LS and their relationship to 5-ASA treatment.

### Descriptive objectives

- To describe tumour occurrence in the right colon, left colon and rectum, as well as the occurrence and location of serrated benign or malignant colorectal neoplasia, in the two treatment groups.
- To investigate whether differences between placebo and 5-ASA in the occurrence of colorectal neoplasia, tumour multiplicity and tumour progression are influenced by cancer history, sex or participant age (participants under 45 years vs 45 years and older).
- To describe the occurrence of colorectal neoplasia, tumour multiplicity and tumour progression for each sublocation in the colon and rectum.
- To determine whether the effects of 5-ASA differ between major LS genotypes.
- To evaluate the effects of 5-ASA treatment on the gut microbiota in LS carriers.
- To evaluate the effects of 5-ASA treatment on gut immunology and mucosal health in LS carriers.
- To assess compliance with 5-ASA treatment in LS carriers.

### Study design

**Figure 1:**
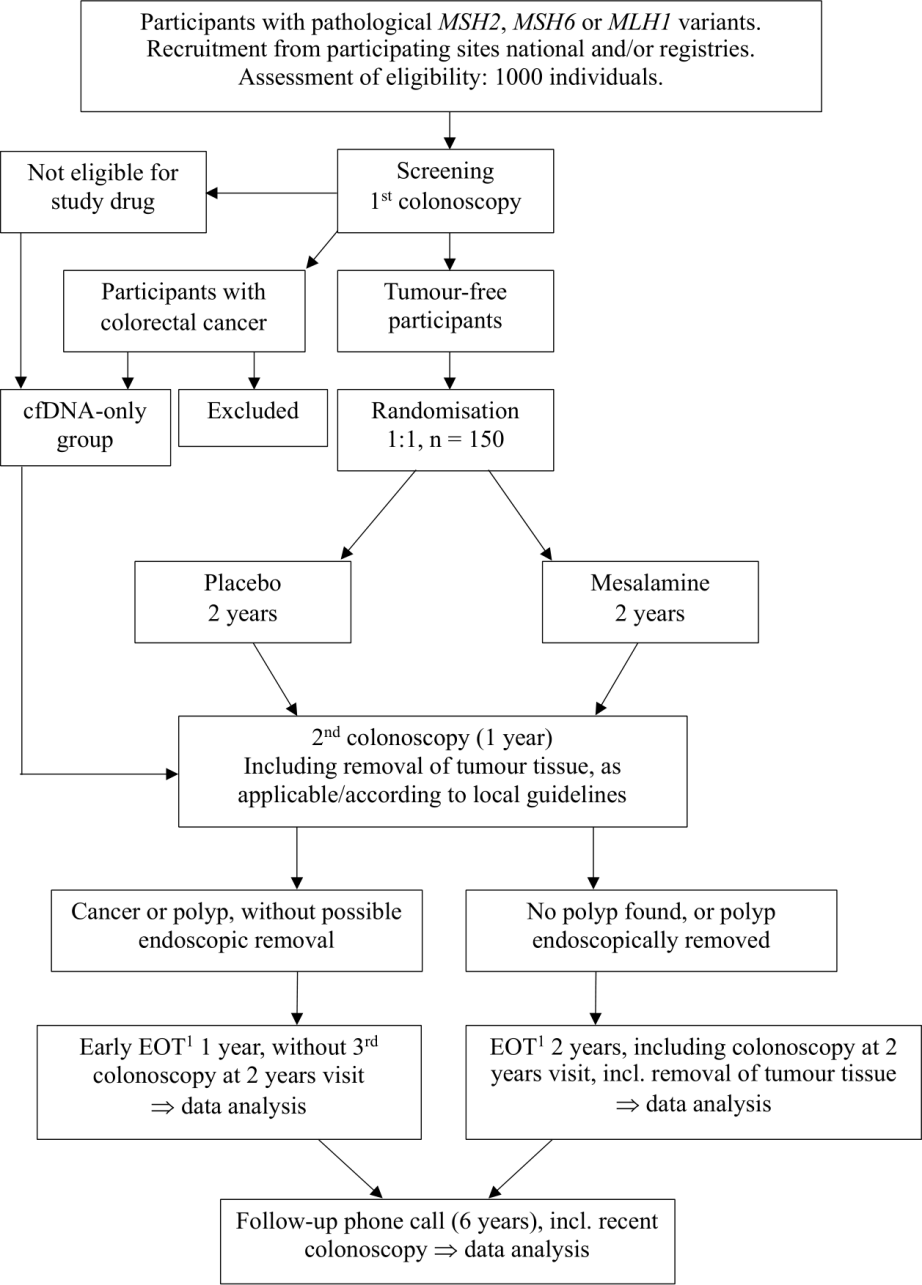
Flowchart, MesaCAPP. ^1^EOT. cfDNA, cell-free DNA; EOT, end of treatment; MesaCAPP, mesalamine for Colorectal Cancer Prevention Programme in Lynch syndrome.

**Figure 2:**
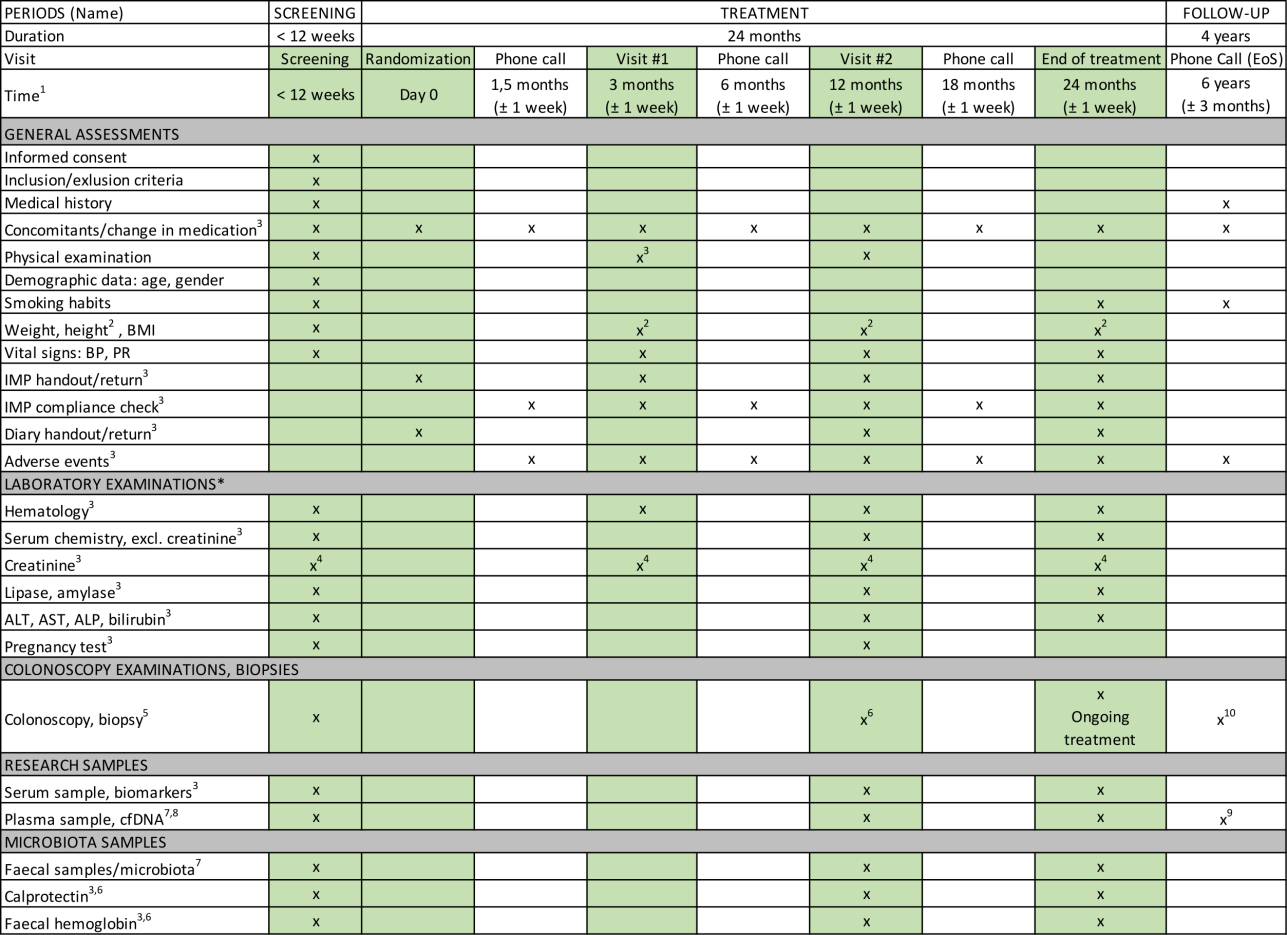
Visit and assessment schedule, MesaCAPP. ^1^After randomisation, ^2^Height will be measured only during the screening phase, ^3^ This is not applicable to participants in the non-treatment cfDNA group, ^4^If elevated, an additional test will be conducted within 1 week, ^5^Additional biopsies will be collected in the event of tumour detection, ^6^In accordance with national/local guidelines, ^7^Only at selected sites, ^8^If any malignancy is detected during the trial, an additional sample should be collected at the time of diagnosis, both before and after tumour excision, ^9^Samples are collected during each colonoscopy throughout the 6-year follow-up period, ^10^Colonoscopies are performed annually in years 3, 4, 5 and 6 after randomisation and the initial colonoscopy. Data from medical records are collected and entered into the eCRF. ALP, alkaline phosphatase; ALT, alanine aminotransferase; AST, aspartate aminotransferase; BMI, body mass index; BP, blood pressure; cfDNA, cell-free DNA; eCRF, electronic case report form; EoS, End of Study; IMP, investigational medicinal product; MesaCAPP, mesalamine for Colorectal Cancer Prevention Programme in Lynch syndrome; PR, pulse rate.

This multicentre, multinational, randomised, double-blind, two-arm, phase II clinical trial will evaluate the efficacy of a daily intake of 5-ASA (2000 mg) compared with placebo in LS carriers over a 2-year treatment period (figure 1). 4 years after the treatment period, follow-up will include a phone consultation and a review of participants’ medical records. Cancer diagnoses and survival data will be collected for up to 18 years post-treatment. Eligibility screening will involve assessing 1000 participants, with 150 participants randomised in a 1:1 ratio into two groups (75 per group). Full registry entries (ClinicalTrials.gov, EudraCT and EU CT) are reproduced in online supplemental table 1. Eligible participants must be tumour-free carriers of an omitted pathogenic variant in a MMR gene, including those with a history of prior endoscopic polyp removal. Participants will be identified through local or national registries and collaborations with study sites. Tumour-free status will be confirmed using high-definition white-light colonoscopy (HDWL). Serum, plasma and stool samples will be collected, along with baseline and 24-month biopsies from normal tissue in the ascending colon, transverse colon and descending colon (figures2  3). Additional biopsies will be taken if polyps are detected.

**Figure 3:**
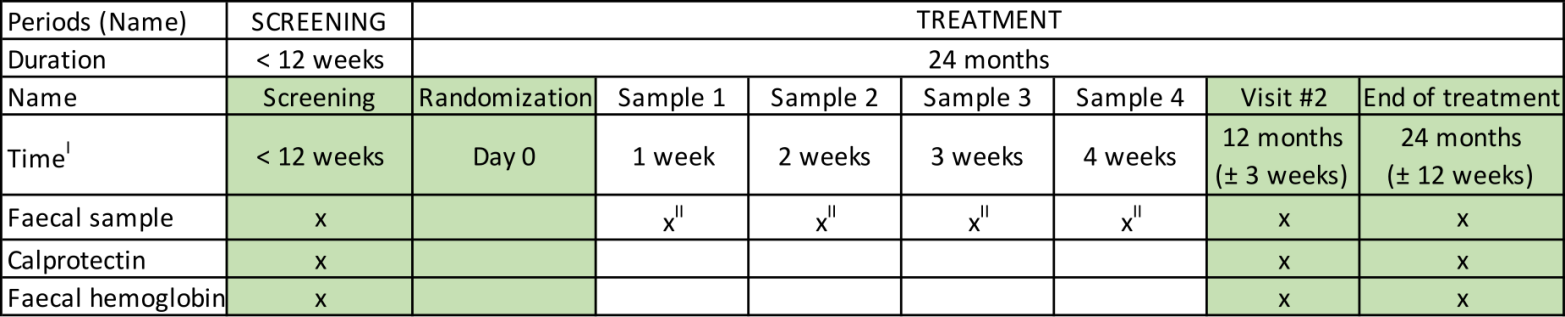
Microbiota sample collection, MesaCAPP. ^I^This is not applicable to participants in the non-treatment cfDNA group, ^II^The first 40 participants who consent to providing these additional samples (optional). cfDNA, cell-free DNA; MesaCAPP, mesalamine for Colorectal Cancer Prevention Programme in Lynch syndrome.

### Allocation, blinding

Randomisation will be conducted via the web-based ALEA system (FormsVision) by the Clinical Trials Office (CTO), Karolinska University Hospital. Participants will be stratified by site and prior CRC history and randomised 1:1 to active treatment or placebo on day 0 using permuted block technique with variable block sizes within strata. Allocation will be concealed within ALEA; the randomisation schedule is held by the CTO and is not accessible to site investigators. Genotype will be addressed analytically: it will be included as a prespecified covariate and effect modifier in the primary model (adjusted for country and prior CRC), with exploratory subgroup reported (see *Statistics, Sample size calculations*). On randomisation, an email confirmation, containing the participant’s unique randomisation number and the blinded study-medication kit number—will be sent to the site. All consortium partners, investigators, site staff, monitors, statisticians and participants will remain blinded until database lock. In an emergency, the investigator may unblind the allocated treatment via ALEA; the reason and timing will be documented, and the participant will remain in the trial for safety follow-up. If interim summaries are produced, they will be arm-masked. Final unblinding for analysis will occur only after all participants have completed the 24-month treatment period and the database has been unlocked.

### Study assessment and procedures

The study includes a 1-month screening period, a 24-month intervention phase and a 4-year treatment-free follow-up. Participants’ medical records will be continuously monitored for up to 20 years following inclusion. Participants with pathogenic or likely pathogenic variants in defined MMR genes will be randomised to receive a once-daily dose of 5-ASA or a visually identical placebo. Eligible participants registered at the clinic will be informed of the study orally or in writing before their next routine visit, with details also available on a dedicated webpage. Following detailed verbal and written information given by the investigator, as well as signed consent, tumour-free LS carriers will be randomised to receive either the medication or placebo, along with a participant-diary. The informed consent form will be filed in the Investigator’s file, with a copy provided to the subject. Participants will attend site visits at 3 and 12 months postrandomisation for renal function assessment (blood creatinine), physical exams, vital signs, diary reviews and medication compliance. Site staff will also conduct phone calls every 6 weeks to monitor adverse events and adherence to the study drug or placebo, aiming for at least 75% compliance. During site visits, empty drug or placebo containers will be exchanged for new medication. An end-of-treatment visit is scheduled 24 months postrandomisation.

Annual or biennial HDWL-colonoscopies will be performed according to each participant’s surveillance protocol to detect colorectal neoplasia. Each procedure will document at least three landmark images (appendiceal orifice, caecum with ileocaecal valve and rectum with scope inversion). Standard quality metrics are recorded in accordance with routine care: the Boston Bowel Preparation Scale, withdrawal time and mandated landmark photographs are recorded.

A follow-up phone visit will occur 6 years after trial initiation to assess the long-term effects of the 2-year 5-ASA treatment. Non-interventional follow-up will continue through review of medical records up to 18 years post-treatment. Participants may withdraw from the study at any time. The sponsor reserves the right to terminate the study at any time. Analyses of gut immunology, microbiota, cell-free DNA (cfDNA) and other biomarkers will be performed using the best-available validated methods at the time of analysis, compared between treatment arms and adjusted for prespecified covariates. The cfDNA cohort is an exploratory substudy; it will not be analysed with respect to the primary endpoint of the parent study and will not contribute to that endpoint. Demographic and disease-related factors will be compared between the cfDNA subgroup and the full study population to assess representativeness.

### Primary endpoint

The occurrence of colorectal neoplasia (both benign and malignant tumours) in the two groups is presented as absolute frequencies and percentages, accompanied by 95% CIs. Logistic regression is used to assess differences in neoplasia occurrence between the active treatment and placebo groups, adjusted for country and prerandomisation cancer history. Treatment effects are expressed as ORs with corresponding 95% CIs.

### Secondary endpoint

The number of any colorectal neoplasia per participant will be compared between groups using analysis of variance, adjusted for country and cancer history before randomisation. If residuals do not meet normality assumptions, a suitable transformation will be applied to achieve normal distribution. Tumour progression across four ordered stages will be compared between groups using a χ² trend test, stratified by country and prerandomisation cancer history, and modelled with ordinal logistic regression. The dependence of treatment effects on CRC history, sex and participant age (<45 years and ≥45 years) will be assessed by modelling interactions between these variables and treatment in the relevant regression models. Safety data will be described and compared between groups in an exploratory analysis.

Extracolonic LS cancers and multiple primaries will be ascertained via medical records/registries, will not alter the primary endpoint and will be reported separately. Their inclusion as covariates will be limited to exploratory sensitivity analyses (prespecified in the statistical analysis plan, SAP), given their postrandomisation nature. Unless otherwise specified, the treatment-policy (intention-to-treat) estimand applies to the secondary endpoints.

### Inclusion criteria

- Proven tumour-free status (including participants who have undergone endoscopic polyp removal) among carriers of a germline pathogenic variant in one of MMR genes, specifically *MLH1*, *MSH2* (including *EPCAM*) or *MSH6*.

- Male or female subjects aged over 30 years.
- Female participants who are postmenopausal for more than 1 year, or females of childbearing potential who are using a highly effective method of contraception (ie, oral hormonal contraceptives, hormone implants, hormone injections, sterilisation, hormonal or copper intrauterine device, sterilised/vasectomised partner or diaphragm combined with a condom, spermicide or birth control pills) or who agree to abstain from heterosexual activity during the treatment period. Females of childbearing potential must present a negative pregnancy test at both screening and randomisation.
- Signed written informed consent was obtained prior to study inclusion.

### Exclusion criteria

- Presence of colorectal neoplasia that is benign but non-removable endoscopically (participants may be included if the adenoma is successfully removed).
- Carriers of germline variants in the *PMS2* gene.
- History of stage III or stage IV CRC.
- Presence of any metastatic disease.
- Regular use of acetylsalicylic acid (ASA or aspirin), defined as daily use of ≥100 mg for more than three consecutive months within the past year.
- Regular use of non-steroidal anti-inflammatory drugs (NSAIDs) or cyclooxygenase-2 inhibitor, defined as daily use for more than three consecutive months within the past year.
- Known hypersensitivity to 5-ASA.
- Participants who have undergone any subtotal or total colectomy.
- Surgery involving the colon or rectum within the previous 6 months.
- Unwillingness or inability to provide informed consent.
- Pregnant or breastfeeding women.
- Participation in another clinical study involving an investigational medicinal product (IMP) within 3 months prior to screening.
- Renal insufficiency, defined as a glomerular filtration rate <30 mL/min/1.73 m².
- Severe liver disease or liver failure, defined as an elevation of liver enzymes to more than three times the upper limit of normal.
- Current or historical serious psychiatric disorders or alcohol/drug abuse that, in the investigator’s opinion, may impact the assessment of IMP safety and efficacy or compromise protocol adherence.
- History of myocarditis or pericarditis.
- Any other severe acute or chronic medical conditions (eg, severe chronic lung disease, including chronic obstructive pulmonary disease or asthma; kidney disease; or heart disease), psychiatric disorders or laboratory abnormalities that may increase the risk of study participation or affect the ability to comply with study procedures or IMP administration. Such conditions, as judged by the investigator, would render the subject inappropriate for study entry.

### Study participants

Consistent with descriptive objectives, we will explore whether 5-ASA effects of mesalamine differ by major LS-genotypes. *PMS2* carriers were excluded a priori given their lower CRC-penetrance, later average age at onset and generally longer recommended colonoscopic surveillance intervals compared with *MLH1*, *MSH2*/*EPCAM* and *MSH6*. Consequently, the expected 24-month event rate among *PMS2* carriers is low; including this low-event stratum would dilute information and yield imprecise *PMS2*-specific estimates (ie, wide confidence intervals), thereby constraining interpretability. After database lock and unblinding, genotype stratified exploratory results and comment on their interpretability, in the light of observed event rates and multiplicity.

### Recruitment and study status

Following the second amendment (21 September 2021), and qualified person approval for IMP release (early 2022), the first participant was enrolled in March 2022. The study remains ongoing; EOT for the last enrolled participant is scheduled for Q3 2027. Originally planned as a European multicentre trial, the study was refocused to a Nordic multicentre trial for logistical reasons. Participating or planned sites are: Sweden-Karolinska University Hospital, Sahlgrenska University Hospital, Uppsala University Hospital, Norrlands University Hospital, Skåne University Hospital and Ersta Hospital; Denmark—Hvidovre University Hospital in Denmark.

### Patient and public involvement

*When and how were patients/public first involved in the research?* The Swedish patient organisation, *Magtarmförbundet*, participated in the study by disseminating informational materials to enhance participant recruitment through information sharing. Regular meetings were conducted with the involved patient representatives throughout the course of the study.

*How were the research question(s) developed and informed by their priorities, experience and preferences?* Patients with LS live with the awareness of a significantly increased risk of developing CRC. For these patients, identifying strategies to reduce this risk is a pressing priority. While the study’s objectives align with these patient priorities and were welcomed by *Magtarmförbundet*, patient representatives were not actively involved in the development of the research questions.

*How were patients/public involved in*

- *The design and conduct of the study?* As noted above, patient representatives were not actively involved in the study design and its conduct.
- *Choice of outcome measures?* As noted above, patient representatives were not actively involved in selecting the outcome measures.
- *Recruitment of the study?* Patients played a role in participant recruitment by disseminating informational materials via patient organisations and by engaging with relatives, given the genetic predisposition associated with LS.

*How were (or will) patients/public be involved in choosing the methods and agreeing plans for dissemination of the study results to participants and linked communities?* Patient forums will be involved in the dissemination phase, offering information and hosting discussion meetings to facilitate knowledge transfer and further spread of findings. The study results will be published through Open Access to ensure broad accessibility and engagement with both participants and the wider community.

### Study completion

The study concludes with the final follow-up call of the last participant, with participant records monitored for 20 years postinclusion. Authorities will be notified within 90 days, or within 15 days if terminated early, with a summary report submitted within 1 year of study completion.

### Data statement section

Data collected at all visits are entered into an interactive form within 5 days after the participant’s visit. The case report form (CRF) has been verified following guidelines established before study onset as detailed in the Data Monitoring Plan (DMP). Maintenance of the study database will be performed by CTO, Center for Clinical Cancer Studies, Cancer Theme, Karolinska University Hospital, Stockholm, Sweden. Data will be collected using the electronic CRF (eCRF) system PheedIt, based on the SAS system provided by Region Stockholm, Stockholm, Sweden, and registered at Center for Clinical Cancer Studies (CTO), Cancer Theme, Karolinska University Hospital, Stockholm, Sweden. For each enrolled subject, an eCRF must be completed and electronically signed by the investigator or designated sub-investigator. Withdrawal reasons and incomplete assessments must be documented and verified in the eCRF by the investigator. Site staff authorised by the investigator will make entries and corrections, which will be monitored by the investigator for accuracy. Following database closure, a printout of completed eCRFs will remain on-site, with originals archived at the sponsor site poststudy. Details on the eCRF will be documented in a separate DMP provided by CTO, Karolinska University Hospital. The investigator must verify the accuracy of all eCRF data entries. The investigator will keep accurate and thorough records to ensure complete documentation of the study and to facilitate data verification, in accordance with International Counsil for Harmonisation’s Good Clinical Practice (ICH-GCP) guidelines. An interim analysis of cfDNA sampling, colonoscopy results, biomarkers and faecal samples will be conducted after two years for the first participant, with data unblinded only to the statistician.

### Statistics, sample size calculation

The statistical design of this trial was originally informed by findings from the CaPP2 trial.^5^ CaPP2 was a 2-year study that employed a 2×2 factorial design to evaluate the effects of resistant starch and aspirin (ASA). After a mean of 27 months on the study drug, 18.9% of participants had developed neoplasia by the second endoscopy. Based on similar inclusion and exclusion criteria, it was estimated that 20% of the placebo group in this study would present with neoplasms after 24 months. A clinically meaningful outcome would be a reduction in neoplasia risk to 7.5%, similar to the reduction observed in the CaPP2 trial. Sample size calculations were based on an assumption of a 20% neoplasia rate in the control group. To detect a reduction to 7.5% in the experimental group (HR 0.375), with a two-sided significance level of 5% and 80% power, a sample size of 236 participants was estimated. At the first interim analysis of the ongoing MesaCAPP (mesalamine for Colorectal Cancer Prevention Programme in Lynch syndrome) study, a higher-than-expected overall neoplasia rate of 50% was observed. This change, while holding other factors constant, would increase the power to detect group differences from 80% to 99.9% with a sample size of 236 participants. A power of 80% would now be achieved with 131 participants. Under more conservative assumptions (HR 0.5) and an increased projected neoplasia rate of 65%, 80% power could still be reached with 101 participants. The trial plans to assess 1000 individuals for eligibility, with an estimated 50% agreeing to participate. However, approximately 75% of those screened are expected to fail to meet the inclusion criteria, based on data from the initial screening period. A 10% drop-out rate is anticipated and accounted for in the sample size calculation. Counts of neoplasia per participant will be analysed using Poisson or negative binomial regression, adjusted for country and prior CRC; overdispersion will be assessed and negative binomial used if indicated. All primary analyses are prespecified; sensitivity analyses assess robustness to missing primary endpoint data (details in the SAP).

Intermittent ASA/NSAID exposure will be recorded for concomitant medication and safety monitoring; the primary analysis includes no prespecified modelling or adjustment for this exposure. If exposure patterns or merging evidence warrant, these data may be included in exploratory or sensitivity analyses.

### Ethics and dissemination

The study protocol was approved by the Swedish Ethical Review Authority (2019–04318) on 23 October 2019, with subsequent amendments approved on 26 April 2021 (2021-01570), 21 September 2021 (2021-04234) and 6 May 2024 (2024-02993-02). Approval was also obtained from the Danish National Medical Research Ethics Committee (2022021008) on 30 March 2022, with an amendment approved on 20 August 2024 (112356).

The protocol published here represents the version, which has been transitioned to the European Clinical Trials Information System in 2024. The trial is registered with ClinicalTrials.gov (NCT04920149) and EudraCT (2019-003011-55).

The investigator will ensure that this study adheres fully to the principles of the Declaration of Helsinki (as amended at the 56th WMA General Assembly, Tokyo, 2008), as well as to the laws and regulations of the country where the clinical research is conducted. Only appropriately trained personnel will participate in the study. All procedures will comply with ICH GCP Guidelines and, if applicable, the Code of Federal Regulations (USA), or other relevant national GCP guidelines. Accordingly, this study follows the Clinical Trials Regulation EU No 536/2014. Compensation for study-related injuries will be provided in accordance with applicable regulations. Study results will be disseminated through publication in peer-reviewed journals and presentations at scientific conferences.

## Supplementary material

10.1136/bmjopen-2025-100082 online supplemental table 1
