# Supplementary material for: Mesalamine for Colorectal Cancer Prevention Programme in Lynch syndrome (MesaCAPP): a multicentre, multinational, randomised, two-arm, double-blind, phase II clinical study with mesalamine or placebo in carriers with Lynch syndrome – a study protocol
Source: BMJ Open. 2025 Nov 9;15(11):e100082. doi: 10.1136/bmjopen-2025-100082 (PMC12598961; doi:10.1136/bmjopen-2025-100082)
Supplement: online supplemental table 1 [file bmjopen-15-11-s001.pdf]

**Data category**	**Information**
Primary registry and trial identifying number	ClinicalTrials.gov NCT04920149
Date of registration in primary registry	2021-06-09
Secondary identifying number	EudraCT: 2019-003011-55, EU CT: 2024-514765-19-01
Source(s) of monetary or material support	Ferring Pharmaceuticals, Lausanne, Switzerland
Primary sponsor	Karolinska Institutet, Stockholm, Sweden
Secondary sponsor(s)	n/a
Contact for public queries	Ann-sofie.backman@ki.se
Contacct for scientific queries	Ann-sofie.backman@ki.se
Public title	MesaCAPP
Scientific title	Mesalamine for Colorectal Cancer Prevention Program in Lynch Syndrome (MesaCAPP)
Countries of recruitment	Sweden, Denmark
Health condition(s) or problem(s) studied	Lynch Syndrome (LS), Colorectal cancer
Intervention(s)	Active comparator: Mesalamine (5-ASA) 2000 mg daily
	Placebo comparator: 2000 mg daily, matching capsules, no active ingredients
Key inclusion criteria	Proven tumor-free (i e. endoscopically removed polyps), carriers with pathogenic variant of MMR-gene: *MLH1* , *MSH2* , *MSH6*
	> 30 years
	Post-menopausal > 1 year or use highly effective contraception if childbearing potential
	Signed, written and informed consent
Key exclusion criteria	Presence of benign colorectal neoplasia that cannot be removed endoscopically
	Carriers of germline variants of *PMS2*
	History of stage III, IV colorectal cancer or any metastatic disease
	Known hypersensitivity to 5-ASA
Studytype	Interventional
	Allocation: randomized, interventional two-arm model
	Parallell assignment masking: double-blind
	Primary purpose: prevention
	Phase II
Date of first enrolment	2022-03-21
Target sample size	150 patients
Recruitment status	Recruiting
Primary outcome(s)	Whether Mesalamine reduces the ocurrence of any colorectal neoplasia, compared to placebo, as detected by any colonosopy within the study's timeframe (24 months ± 3 months)
Key secondary outcomes	Whether Mesalamine reduces the ocurrence of any colorectal neoplasia or tumour progression, compared to placebo, at defined timepoints
	Safety regarding Mesalamine in LS-carriers
	Investigate metabolic, inflammatory and genomic biomarkers associated to LS-carriers, and in relation to Mesalamine-treatment
