# Supplementary material for: Mesalamine for Colorectal Cancer Prevention Programme in Lynch syndrome (MesaCAPP): a multicentre, multinational, randomised, two-arm, double-blind, phase II clinical study with mesalamine or placebo in carriers with Lynch syndrome – a study protocol
Source: BMJ Open. 2025 Nov 9;15(11):e100082. doi: 10.1136/bmjopen-2025-100082 (PMC12598961; doi:10.1136/bmjopen-2025-100082)
Supplement: Supplementary file 2 [file bmjopen-2025-100082.reviewer_comments.pdf]

PEER REVIEW HISTORY

BMJ Open publishes all reviews undertaken for accepted manuscripts. Reviewers are asked to complete a checklist review form and are provided with free text boxes to elaborate on their assessment. These free text comments are reproduced below.

ARTICLE DETAILS

Title (Provisional)

Mesalamine for Colorectal Cancer Prevention Program in Lynch Syndrome - MesaCAPP. A multicenter, multinational, randomized, two-arm, double-blind, phase II clinical study with Mesalamine or placebo in carriers with Lynch Syndrome.

Authors

Backman, Ann-Sofie; Frank, Alexander; Lindberg, Lars Joachim; Ljungman, David; Silander, Gustav; Gustafsson, Rita J; Bozsó, Tünde; Schmidt, Peter T; Ingre, Michael; Mittlbock, Martina; Löwbeer, Christian; Marsal, Jan; Lindblom, Annika; Tham, Emma; Therkildsen, Christina; Gasche, Christoph; The International MesaCAPP Study Group, .

VERSION 1 - REVIEW

**Reviewer**	**1**
**Name**	**Chambuso, Ramadhani**
**Affiliation**	**Harvard T H Chan School of Public Health**
**Date**	**29-Aug-2025**
**COI**	**None**

I have read this well-written work with a very high interest. Safe chemoprevention for LS is ideal. While the concept of aspirin chemoprevention in LS is well known, the novelty of this clinical trial is testing mesalamine as an MSI-targeted, well-tolerated alternative for lifelong LS chemoprevention because aspirin does not affect MSI, whereas mesalamine has been shown to improve replication fidelity and reduce MSI in cell and mouse models.

(MesaCAPP tested mesalamine (5-ASA, 2000 mg daily, 24 months) versus placebo in a randomized study of verified LS carriers, across European sites, with primary outcome colonoscopic colorectal neoplasia.

Known fact: 5-ASA is well-tolerated and may reduce microsatellite instability.

Unknown fact: preventive efficacy and effect modifiers (age, sex, prior cancer).

Existing gap: no randomized chemoprevention trial in LS.

Critical need: a scalable, low-toxicity agent; this trial evaluates efficacy, safety, and mechanistic signals to inform phase III.

Specific views and comments

i) How will randomization/analysis stratify by germline gene (MLH1, MSH2/EPCAM, MSH6) and justify exclusion of PMS2, given gene-specific CRC risks and phenotypes that may modify chemopreventive effect?

ii) Which tissues/timepoints and assays will quantify MSI/replication fidelity (PCR panel vs NGS; normal mucosa vs adenomas), to test the hypothesized 5-ASA effect on MMR-defect–driven MSI? Can this information be added in the protocol?

iii) Given that the primary outcome is neoplasia at colonoscopy to 24 months, how are colonoscopy quality metrics (prep, withdrawal time, right-colon techniques) standardized, especially for right-sided/serrated LS lesions? Also, what about non-colorectal LS cancers??or co-existing of multiple cancers from other body sites? CRC with endometrial cancer.

iv) How will intermittent low-dose ASA/NSAID use be captured (beyond excluding regular users) and modeled, given potential interaction with MMR-driven tumorigenesis and adherence?

v) With 150 randomized and a 24-month window, what baseline LS neoplasia rates, dropout, and surveillance intensity inform power, especially as annual colonoscopies continue in later follow-up?

vi) Optional fecal microbiome, calprotectin, and fecal hemoglobin sampling risks selection bias; how will these be linked to MMR-defect immunobiology and integrated with the no-treatment cfDNA cohort?

VERSION 1 - AUTHOR RESPONSE

Peer-review comments:

i) How will randomisation/analysis stratify by germline gene (MLH1, MSH2/EPCAM, MSH6) and justify exclusion of PMS2, given gene-specific CRC risks and phenotypes that may modify chemopreventive effect?

Thank you for this important comment. Randomisation strata were implemented via ALEA (FormsVision) and fixed *a priori* as site and prior colorectal cancer (CRC) history. Introducing gene (*MLH1*, *MSH2/EPCAM*, *MSH6*) as a new randomisation stratum mid-trial would require protocol amendment, ethics re-approval, system reprogramming, and additional site retraining, thereby risking operational disruption and potential selection bias. Accordingly, without altering the protocol, gene-specific effects will be addressed analytically: genotype will be included as a prespecified covariate and effect modifier in the primary model, adjusted for country and prior CRC (Study protocol, v10.0, 11 April 2024, p. 51) with subgroup estimates. For count outcomes, we will use Poisson or negative binomial models; for binary outcomes, we will use logistic regression. As per the descriptive objectives, we will

examine whether 5-ASA effects differ by major Lynch Syndrome (LS) genotypes. Exclusion of *PMS2* remains justified given its lower CRC penetrance, later age at onset, and generally longer recommended colonoscopic surveillance intervals, compared with those recommended for *MLH1*, *MSH2*/*EPCAM*, *MSH6*. As a consequence, the 24-month event yield among *PMS2* carriers is expected to be low. Inclusion of this low-event stratum would reduce information content of the trial, and yield imprecise *PMS2*-specific efficacy estimates (i.e., wide confidence intervals (CI)), thereby constraining interpretability. Upon completion and unblinding, we will present genotype-stratified exploratory results and discuss their interpretability in the light of observed event rates and multiplicity considerations.

ii) *Which tissues/timepoints and assays will quantify MSI/replication fidelity (PCR panel vs NGS; normal mucosa vs adenomas), to test the hypothesised 5-ASA effect on MMR-defect–driven MSI? Can this information be added in the protocol?*

We agree this is important and will address it at the time of analysis. It is stated (protocol, p. 52) that analyses of gut immunology, microbiota, cfDNA, and biomarkers will be performed using the best-available validated methods at the time of analysis, compared between treatment arms, and adjusted for prespecified covariates. Given Phase II lead-times, prespecifying a single assay platform years in advance, risks lock-in to superseded methods; full assay details (panels, Quality Control (QC) treshholds) will be finalised in Standard Operating Procedures (SOP)/Statistical Analysis Plan (SAP) before unblinding. Anticipated methodological and technical advances, evolving conditions for conducting medical research, resource availability, and grant allocation may all influence the final choice of methods.

iii) *Given that the primary outcome is neoplasia at colonoscopy to 24 months, how are colonoscopy quality metrics (prep, withdrawal time, right-colon techniques)standardised, especially for right-sided/serrated LS lesions? Also, what about non-colorectal LS cancers??or co-existing of multiple cancers from other body sites? CRC with endometrial cancer.*

Within the current protocol, colonoscopy quality is standardised via high-definition white-light colonoscopy (HDWL) with documented Boston Bowel Preparation Scale (BBPS) scores, withdrawal time, and required landmark photographs: appendiceal orifice, ileocaecal

valve, rectal retroversion (protocol, p. 37). To support detection of right-sided and serrated lesions, without protocol change, sites follow existing QC procedures; recognised adjunct technics may be used per local SOPs and are recorded, but not mandated. Introducing new mandated procedures mid-trial is impracticable; accordingly, the protocol relies on existing SOPs and documentation to contextualise performance. In addition, central oversight uses site-level dashboards derived from existing data, including adenoma detection rate (ADR) and sessile serrated lesion (SSL) detection indicators.

Analytically, the primary model is logistic regression adjusted for site and prior CRC-history. Sensitivity analyses will (1) adjust for right-segment BBPS and withdrawal time, (2) exclude examinations with inadequate right-colon preparation (right segment, BBPS <2), and (3) address site heterogeneity via cluster-robust standard errors or a site random effect. If no eligible colonoscopy is available, the endpoint will be handled under the prespecified multiple-imputation framework [1-3], with robustness assessed using conservative bounding analyses.

Extra-colonic LS cancers and multiple primaries are captured via records/registries, do not alter the primary endpoint, and will be included as covariates in sensitivity analyses. The estimand reflects the effect of assigning 5-ASA vs placebo, irrespective of interruptions, co-medications or missed visits (intention-to-treat rather than per-protocol).

iv) How will intermittent low-dose ASA/NSAID use be captured (beyond excluding regular users) and modeled, given potential interaction with MMR-driven tumorigenesis and adherence?

Regular ASA/NSAID/COX-2 users are excluded per protocol (protocol, pp. 23-24).

”Regular use” is defined as daily ASA ≥100 mg or daily NSAID/COX-2 intake, for more than three consecutive months within the past year. Intermittent exposure (start/stop dates, dose, duration) will be obtained from self-report and site records, and entered into the electronic Case Report Form (eCRF). ASA/NSAID use will be captured using established instruments, including patient diaries, six-weekly safety/adherence calls, and on-site medication reviews. While detailed information on intermittent exposure will be collected to monitor concominant medication use and ensure participant safety, no pre-specified statistical modelling or adjustment for such exposure is planned in the primary analysis. However, these data may be considered for exploratory or sensitivity analyses, should exposure patterns or emerging evidence warrant further investigation.

v) *With 150 randomized and a 24-month window, what baseline LS neoplasia rates, dropout, and surveillance intensity inform power, especially as annual colonoscopies continue in later follow-up?*

The primary endpoint (protocol, pp. 19-20) is assessed at end of treatment (EOT: 24 months + 12 weeks). Colonoscopies performed thereafter contribute to end-of-study (EOS) reporting and do not affect the EOT analysis. Surveillance follows standard care (annual/biennial) and most participants are expected to undergo ≥ 1 qualifying colonoscopy within the EOT window. Quality metrics, HDWL, BBPS-scoring, withdrawal time, and mandated landmark photography - are routinely recorded and will inform sensitivity analyses. Adjunctive techniques may be used per local SOPs and are documented. The study remains powered (study protocol, pp. 50-51) at n=150 (1:1) under a treatment-policy (intention-to-treat) estimand, using stratified logistic regression (strata: country; covariate: prior colorectal cancer). CaPP2-informed original assumptions, anticipated a ~20% 24-months control neoplasia rate, requiring n=236 to detect a reduction to 7.5% (HR 0.375, two-arm, α=0.05, 80% power). A prespecified, arm-masked interim look indicated a higher overall neoplasia rate (~50%), increasing the information available for estimation. Under these conditions, 80% power corresponds ~n=131, rendering n=150 robust. Genotype is handled analytically (covariate/interaction); subgroup estimates will be reported as exploratory with 95% CIs. Missing primary outcomes will be addressed via the prespecified multiple-imputation framework according to established guidelines [1-3], with conservative bound-based sensitivity analyses. Primary inference is based solely on in-window colonoscopies.

vi) *Optional fecal microbiome, calprotectin, and fecal hemoglobin sampling risks selection bias; how will these be linked to MMR-defect immunobiology and integrated with the no-treatment cfDNA cohort?*

As specified (protocol, p. 52), analyses of gut immunology, microbiota, cfDNA and other biomarkers will be performed, compared between treatment arms, and adjusted for prespecified covariates. The cfDNA cohort is an exploratory substudy, will not be analysed with respect to the primary endpoint of the parent study and will not contribute to that endpoint. We will compare demographic and disease-related factors between the cfDNA

subgroup and the full study population to assess representativeness. Finally, it is recognised that optional fecal sampling may introduce selection bias; participation will be described, and analytic remedies (e.g., inverse-probability weighting and multiple imputation [1-3]) prespecified in the SAP.
